# Comparative Proteomic Analysis of Gallbladder Bile Proteins Related to Cholesterol Gallstones

**DOI:** 10.1371/journal.pone.0054489

**Published:** 2013-01-17

**Authors:** Diancai Zhang, Jianbin Xiang, Liying Wang, Zhibin Xu, Lidong Sun, Feng Zhou, Xiliang Zha, Duan Cai

**Affiliations:** 1 Department of General Surgery, the First Affiliated Hospital, Nanjing Medical University, Nanjing, People’s Republic of China; 2 Department of General Surgery, Huashan Hospital, Fudan University, Shanghai, People’s Republic of China; 3 Department of Surgery, Shanghai Medical College, Fudan University, Shanghai, People’s Republic of China; 4 Key Laboratory Glycoconjugate Research, Ministry of Health, Department of Biochemistry and Molecular Biology, Fudan University, Shanghai, People’s Republic of China; University of Bari & Consorzio Mario Negri Sud, Italy

## Abstract

**Background:**

Nucleation of cholesterol monohydrate crystals following the aggregation and fusion of cholesterol-enriched vesicles is a critical procedure in the formation of cholesterol gallstone. Biliary proteins play important roles in the process. It is inefficient to screen pro-nucleating or anti-nucleating proteins with routine physiochemical techniques, by which we discovered several pro-nucleating proteins.

**Methodology/Principal Findings:**

Based on comparative proteomic technologies, we investigated the differentially expressed proteins between the cholesterol gallstone and control groups, and between the vesicular phase and micellar phase. There are 401±75 spots detected on the cholesterol gallstone group and 389±94 spots on the control group gels, 120±24 spots detected on vesicular phase and 198±37 on micellar phase gels, and accordingly 22 and 8 differentially expressed proteins were identified successfully, respectively. Three of them, HSA, Profilin and Retinol Binding Protein, were validated by Western blot.

**Conclusion/Significance:**

Some of the identified proteins are in good agreement with proteins reported to be involved in the gallstone formation before. The information from this study might provide some important clues to uncover the key proteins involved in the formation of cholesterol gallstone.

## Introduction

Gallstone disease is one of the most prevalent gastrointestinal diseases with a substantial burden to health care systems that is supposed to increase in aged populations at risk [1]–[3]. Diehl reported that cholesterol gallstones account for 80–90% of the gallstones found at cholecystectomy in Western societies [4]. Etiology and pathogenesis of cholesterol gallstones are still not well defined, but three mechanisms are of major importance, cholesterol supersaturation, gallbladder hypomotility and kinetic, pro-nucleating or anti-nucleating factors [1], [5]. The multifactorial pathogenesis of cholesterol gallstone has been widely accepted, and the relative significance of various factors remains to be clarified. But, biliary proteins might involve in the above three mechanisms, such as regulation of gallbladder motility and alteration of nucleating activity.

The cholesterol can be dissolved in gallbladder bile mainly due to distinct carriers: mixed micelles consisting of bile salts, phospholipids and cholesterol and vesicles containing phospholipids and cholesterol [6]. It is widely accepted that nucleated cholesterol originates from cholesterol-enriched vesicles [7]. Any agent that affects vesicle aggregation therefore affects collisions of prevailing clusters, and hence the rate of nucleation. Accordingly, the characterization of proteins associated with vesicles or mixed micelles in bile is of particular interest, since these might affect the solubility of cholesterol and following the formation of cholesterol gallstone.

Since the first report of the presence of pro-nucleating activity proteins in bile [8], several proteins are known or suspected to influence the kinetics of cholesterol nucleation and classified as either pro-nucleating or anti-nucleating agents [9], [10]. In recent years, several publications have marshalled experimental evidence arguing against a role of most of biliary proteins in cholesterol gallstone formation. Their results showed that excess biliary proteins been occurred secondary to supersaturation rather than as a primary defect causing cholesterol crystallization [11], [12]. None of these nucleation factors has been unequivocally linked to either cholesterol gallstone disease. The failure can be explained by the fact that formation of cholesterol gallstone is an extremely complex process under control of numerous genetic and environmental factors [13]. However, since the complex components of gallbladder bile interfere with the analysis of biliary proteins, it is not perfect that nucleating activity proteins were sifted and identified by routine physiochemical technologies in the past. Perhaps, many proteins, playing important roles in formation of cholesterol gallstone, cannot be detected with these technologies.

He et al counted 70 and 7 spots in 2-DE patterns of bile from patients with cholesterol gallstones and pigment gallstones, 59 and 471 spots from vesicular and micellar phases, respectively. They did not analyze the different proteins by MS further [14], [15]. Kristiansen et al identified 87 unique proteins in pathologically changed bile fluid obtained from a cholangiocarcinoma patient [16]. Zhou et al made a large-scale (218 proteins) identification of biliary proteins of a cholesterol gallstone patient during cholecystectomy [17]. But to our knowledge, there is no literatures elucidated about the differentially expressed proteins in gallbladder bile between cholesterol gallstone and control groups or between vesicular phase and micellar phase with 2-DE and MALDI-TOF MS and tandem TOF/TOF MS.

In recent years, proteomics has provided unparalleled information in the understanding of pathogenesis of kinds of diseases. System analysis performed at the protein level has the advantage of being closest to their function. In this study, we employed comparative proteomic approaches to investigate the differentially expressed proteins associated with gallbladder bile from cholesterol cholelithiasis patients and control ones, and mixed micelles and vesicles in gallbladder bile from patients with cholesterol gallstones. We attempt to explore the key protein which might relate to cholesterol gallstone formation. It might help us cultivate a better understanding of the pathophysiologic molecular mechanisms involved in the formation of cholesterol gallstone.

## Methods

### Gallbladder Bile Samples

Thirty-one patients with symptomatic cholesterol gallstone disease (mean age: 45.7±6.9 years, 9 male/22 female) scheduled for elective cholecystectomy were included in the present study. There were more than two stones in the gallbladder of every subject. Patients with a diagnosis of hepatic dysfunction, acute cholecystitis, or biliary ducts obstruction were excluded. Cholesterol content of dry weight as determined by chemical analysis was more than 70% in all the cases. Gallbladder bile samples were obtained during surgery as described by Strasberg et al [18] and stored at −80°C until processed. Gallbladder bile acquired from 8 liver transplantation donors (mean age: 33.4±10.4 years, 2 male/6 female), who were free of gallstone, were included in the control group. The clinical characteristics of cholesterol gallstone patients and controls are showed in Table 1. Inclusion of patients in this study was approved by the Institutional Ethical Committee of the First Affiliated Hospital of Nanjing Medical University, China. The study complies with the Declaration of Helsinki, 1995. The subjects gave full written informed consent. Some samples in this study from minors, we obtain written informed consent from guardians on the behalf of the minor participants. The patient anonymity has been preserved. This institutional review board specifically approved this study.

**Table 1:** Clinical characteristics of cholesterol gallstone patients and controls.

	Gallstone patients(n = 31)	Control subjects(n = 8)	p
Gender(M:F)	9∶22	2∶6	>0.05
Age(yr)	45.7±6.9	33.4±10.4	<0.05
ALT(IU/L)	28.5±12.1	30.7±8.4	>0.05
AST(IU/L)	25.7±9.3	26.5±9.2	>0.05
BMI(kg/m^2^)	24.7±3.1	22.4±4.6	<0.01
CSI	1.37±0.45	1.02±0.23	<0.01

a) Continuous variables are presented as mean ± standard deviation.

b) ALT = Alanine aminotransferase; AST = Aspartate aminotransferase; BMI = Body Mass Index; CSI = cholesterol saturation index.

Bile fluid samples were centrifuged at 15 000 g for 15 min at 4°C to remove debris and cells as a preliminary separation. They were then dialyzed against Tris-base (0.01 M, pH 7.54) for 48 h at 4°C. After the bile samples were centrifuged at 15 000 g for 45 min, the stratum intermedium was mixed (v/v 1∶5) with 0.1% acetic acid in a 50∶50 mixture of acetone and ethanol at −20°C overnight. The supernatant was removed by centrifugation at 15 000 g for 30 min. Washed with cold acetone, the pellets were then lyophilized and dissolved in a buffer containing 7 M urea, 2 M thiourea, 4% CHAPS, 40 mM Tris, 65 mM DTT, 2% Pharmalyte and 1 mM PMSF. The protein concentration was determined according to the Bradford method with BSA as the protein standard. All the samples were stored at −80°C prior to further analysis.

### Isolation of Various Cholesterol-carried Phases

Various cholesterol-carried phases were isolated using the density gradient centrifugation method described by Miquel et al [19] with some modifications. Gallbladder bile was centrifuged at 12 000 g for 10 min at 4°C, filtered through 0.22 µm micropore filters (Whatman Ltd, Maidstone, Kent, U.K.). Metrizamide (13% w/v; Sigma, St. Louis, MO, USA) was directly dissolved in bile. Then the bile mixture was centrifuged at 59 000 rpm for 4 h at 4°C in a SW 60 Ti Swinging Bucket Rotor (Beckman Instruments, Palo Alto, CA, USA). The top opalescent vesicular and bottom micellar fraction was obtained by fine needle. The integrity of the two fractions was confirmed by transmission electron microscopy. Then the two objective fractions were processed through the same procedures as for crude gallbladder bile samples.

### One-dimensional SDS-PAGE Electrophoresis

To validate the effect of samples processing and observe one dimension protein profiles, aliquots (2 µg) of protein samples were re-solubilized with sample buffer (50 mM Tris, 2% SDS, 10% glycerol, 0.1% bromophenol blue, 5% beta-mercaptoethanol, pH 6.8). Gels were stained with silver nitrate [20].

### 2-DE

The protocol of 2-DE was followed as described by Görg with modification [21], [22], using the IPGphor IEF System and Hoefer SE 600 (Amersham Biosciences, Uppsala, Sweden). Total proteins (250 µg) of each sample were run in IEF using a 13 cm pH 3–10 NL IPG strips (Amersham Biosciences). After active in-gel rehydration under a low voltage of 30 V at 20°C for 12 h, IEF was performed using the following parameters: 100 V for 1 h, 200 V for 2 h, 500 V for 1 h, 1000 V for 1 h, 8000 V (gradient) for 0.5 h, and finally 8000 V for a total of 24 000 Vh. Then, the IPG strips were immediately reduced and alkylated with buffer I (6 M urea, 30% glycerol, 2% SDS, 1% DTT) and then buffer II (6 M urea, 30% glycerol, 2% SDS, 2.5% iodoacetamide) each for 15 min. Vertical SDS-PAGE was run with laboratory-made homogeneous acrylamide gel (12.5%T, 3%C). SDS-PAGE was performed for 30 min at a constant voltage of 40 V per gel and then 100 V per gel until the bromophenol blue reached 1 mm from the bottom of the gels.

### Protein Visualization and Image Analysis

The gels were visualized by silver staining using the protocol of Mortz et al [20]. Gels were also run with the same samples yet more loading amount (1 mg). These gels were stained with CBB R-250 for preparative gels for MS detection. Subsequently, the 2-DE gels were scanned using ImageScanner™ (Amersham Biosciences). Image data were analyzed using ImageMaster™ 2D Platinum version V 5.0 software (Amersham Biosciences). The spot automatic detection function was used for all group comparisons using the same parameters. Groups were matched automatically and corrected manually if necessary. Differences in protein expression were identified using the relative volume (%Vol) option of the software. Statistical analysis was performed using the Student’s *t*-test between different groups.

### MALDI-TOF-MS and Database Search

The spots were excised from the gels and in-gel digested with sequencing grade trypsin (Promega, Mannheim, Germany). Firstly, the excised gel spots were destained with 50% ACN/50 mM (NH_4_)_2_CO_3_ and washed in Milli-Q water. The spots were then soaked in 100% ACN for 5 min to dehydrate the gels. After being dried with Speed-Vac (SPD 111 V; Savant Instruments, Holbrook, NY, USA) for 20–30 min, the gels were rehydrated with about 15 µL cold trypsin solution (12.5 ng/µL), and incubated at 37°C for 16–24 h. The gels were extracted for peptides in 25–50 µL of 50% ACN/0.1% TFA for 30–60 min, and the two extracts were combined and completely dried with Speed-Vac. The peptide eluate (0.5 µL) was applied to a MALDI target and overlaid with 0.5 µL CHCA (10 mg/mL in 50% ACN, 0.1% TFA). MALDI-TOF MS and tandem TOF/TOF MS was performed on a 4700 MALDI-TOF/TOF Proteomics Analyzer (Applied Biosystems, Foster City, CA, USA). MASCOT search engine (www.matrixscience.com) was used to identify proteins.

### Western Blot

Three of the differentially expressed proteins, HSA, Profilin (PFN), and Retinol binding protein (RBP) were further validated by Western blot analysis. Mouse mAb against HSA was from Invitrogen (Carlsbad, CA, USA), Mouse pAb against human PFN from Alexis Biochemicals (Lausanne, Switzerland), and mouse mAb against RBP from Abcam(Cambridge, MA, USA). The immune complexes were visualized by chemiluminescence using the ECL kit (Amersham Biosciences Piscataway, NJ, USA). We did not find suitable internal standard in gallbladder bile to be used in the study. The film signals were digitally scanned and then quantified using TotalLab 2.01 (Nonlinear Dynamics Ltd, USA).

## Results

### Sample Preparation and SDS-PAGE Profiles

Gallbladder bile is a complex system mainly composed of water, inorganic ions, conjugated bile salts, phospholipids, cholesterol, bilirubin and proteins. The high amounts of lipids, bilirubin and bile salts in crude bile or different cholesterol-carried phases, which interfered with our analysis, were removed by dialysis and ultracentrifugation. These procedures provided us with satisfactory SDS-PAGE maps without the smearing of kinds of contaminants in bile. Most of the protein bands of cholesterol cholelithiasis and control groups are between 47.5 kDa and 83 kDa. The abundance of major expressed proteins is higher in experiment group than in control group. Several proteins with low and high molecular weight differentially expressed between two groups. The protein maps of different cholesterol-carried phases indicate that a very low amount of proteins is associated with vesicular phase. These results show that gallbladder bile protein profiles of SDS-PAGE from different groups and from different cholesterol-carried phases are unique and well-defined.

**Figure 1:**
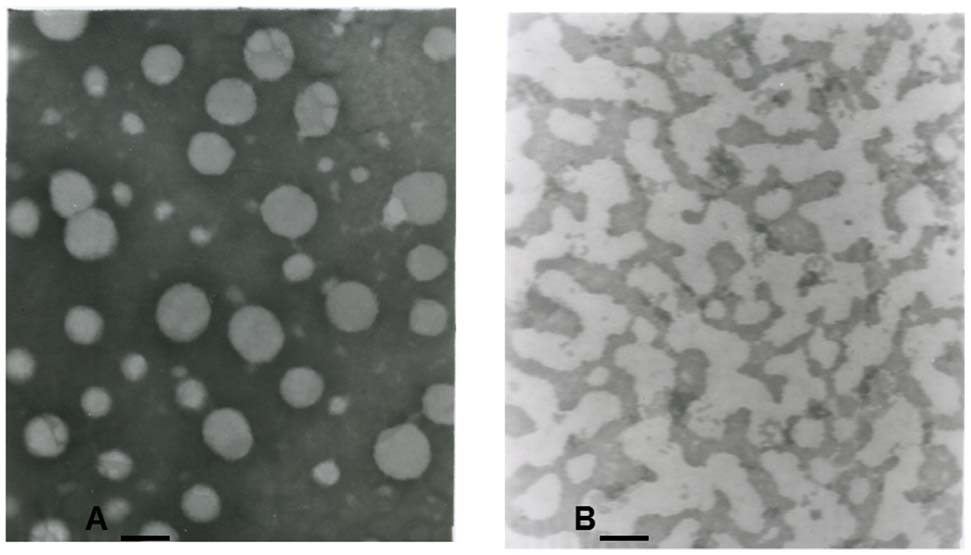
Transmission electron microscopy of vesicular and micellar phases. Bar = 100 nm, JEM-1200Ex. A: General view of cholesterol-phosphatide vesicle. B: General view of micellar phase.

Figure 1 is representative topographic images of transmission electron microscopy of different cholesterol-carried phases. Many vesicles, most of which range in size from 10 nm to 100 nm, are randomly distributed in the vesicular phase. There also appear to be aggregated vesicles, which may have been present in native bile or arose from the processing. No vesicles can be found in the micellar phase obtained from ultracentrifugation. The density gradient centrifugation procedure for isolation different cholesterol-carried phases is validated through the morphological confirmation of transmission electron microscopy.

### Comparative Proteomic Analysis between the Gallbladder Bile of Control and that of Cholesterol Cholelithiasis Groups

**Figure 2:**
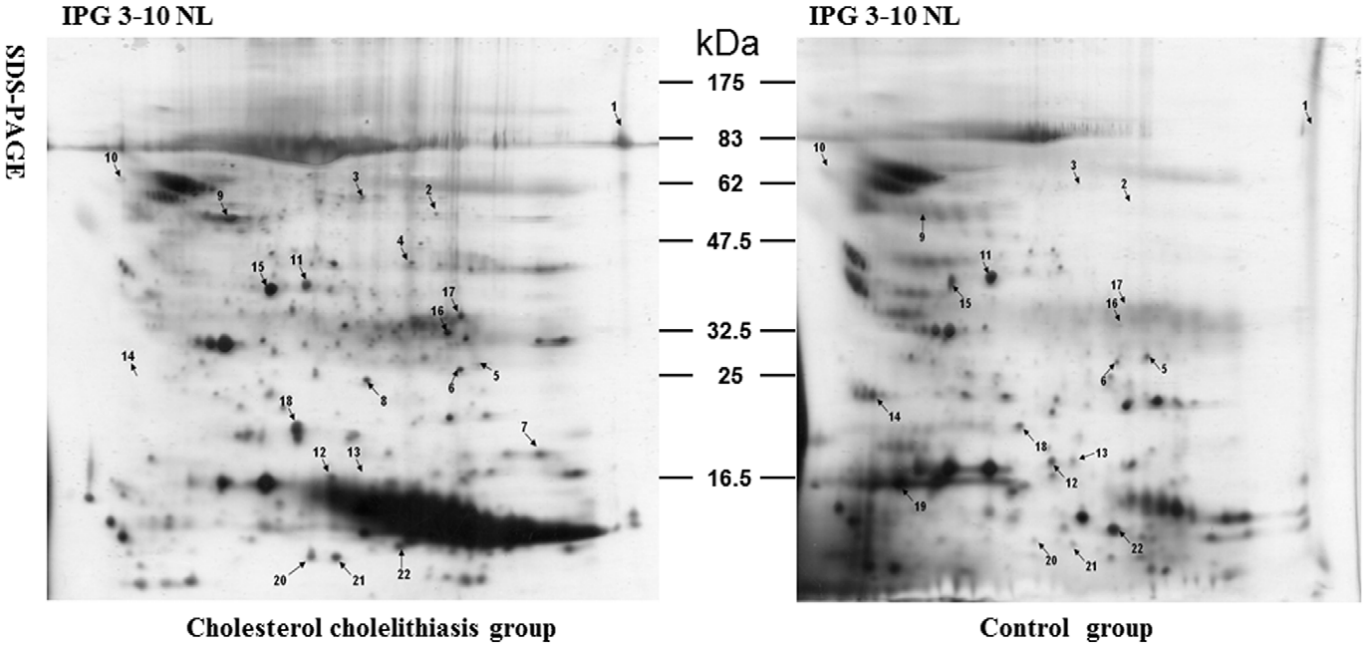
Representative 2-DE profiles of gallbladder bile proteins from cholesterol cholelithiasis group and control group. The proteins (250 µg) were subjected to 2-DE system (first dimension, IPG strip, pH 3–10 NL, 13 cm; second dimension, 12% SDS-PAGE). Protein spots were visualized by silver staining and analyzed by ImageMaster™ 2-D Platinum software. The spots marked with arrows indicate the distribution of up-regulated or down-regulated proteins that were successfully identified by MALDI-TOF. The numbers between the two images show the molecular marker (kDa). Annotations in the gels refer to the Spot No. are in Table 2.

To reveal the different proteins in gallbladder bile from cholesterol cholelithiasis patients and control ones, we compared the protein profiles of two groups. There were 401±75 (n = 26) spots detected on the experimental and 389±94 (n = 8) spots on the control gels. The number of spots matching experimental with control group were 216±32 (54.7%) (Figure 2). We focused on 34 spots that showed the most abundant of the differential expression and statistically significant (Student’s *t*-test, p<0.05). Then these spots were cut from CBB staining gels to perform MS identification. Figure 2 showed 22 spots were identified successfully according to their peptide mass fingerprints.

**Table 2:** Differentially expressed proteins of gallbladder bile between cholesterol cholelithiasis group and control group identified by MALDI-TOF MS.

SpotNo.a)	Protein Descriptionb)	Accession No.c)	Theoretical Mr (Da)/pId)	Matched peptidese)	Protein coverage (%)f)	Protein Scoreg)
1	Chain A, The Ga Module Complexed With Human Serum Albumin	55669910	67173.9/5.57	39	69	660
2	Immunoglobulin Gamma 2 Heavy Chain Constant Region	25987833	36418/7.51	11	48	204
3	Chain A, Human Serum Albumin	4389275	65992.7/5.69	22	33	301
4	Aldo-keto Reductase Family 1, Member A1	5174391	36549.9/6.32	19	54	350
5	Hypothetical Protein	29789193	47863.5/8.28	3	12	79
6	Chain B, Recombinant Hemoglobin	2982014	15903.3/7.13	10	77	223
7	Variable Immunoglobulin Anti-TTd Light Chain	1834499	11609.8/8.7	7	73	131
8	Beta-Actin	14250401	40978.4/5.56	18	59	340
9	Mitochondrial ATP Synthase, H+ Transporting F1complex Beta Subunit	89574027	46173.2/5.03	19	55	586
10	Calreticulin Precursor Variant	62897681	42890.1/4.3	11	25	152
11	Chain A, Beta-Actin-Profilin Complex	576368	41800.7/5.21	18	50	341
12	Chain A, Lipid-Free Human Apolipoprotein A-I	90108664	28061.5/5.27	27	84	443
13	Proapolipoprotein	178775	28943.9/5.45	20	66	284
14	Ttropomyosin	438878	28988.7/4.75	23	50	270
15	Alpha1-acid Glycoprotein	757907	42000.3/4.96	10	45	198
16	Fibrinogen Beta Chain Precursor	20178280	50762.9/7.95	10	19	109
17	Immunoglobulin Heavy Chain	1321859	70478.6/5.01	17	23	175
18	Hypothetical Protein	2135416	30162/5.07	4	11	76
19	Chain A, Lipid-Free Human Apolipoprotein A-I	90108664	28061.5/5.27	31	90	511
20	Chain A, The Transthyretin Mutant	31615374	13717.9/5.35	4	14	80
21	Chain B, Human Retinol Binding Protein	4558176	12994.5/5.53	10	77	384
22	Apolipoprotein A-II Precursor	114000	8707.9/5.05	6	54	189

a) Spot number corresponds to the spot number on Fig. 2.

b) Protein description refers to the name of each matched protein in NCBInr database.

c) Accession number is recorded as a reference for the identification in NCBInr database.

d) Theoretical Mr/pI means theoretical molecular weight and iso-electric point of the matched protein.

e) Matched peptide refers to the number of peptide matched to the candidate protein.

f) Sequence coverage is percent of identified sequence to the complete sequence of the candidate protein.

g) Spots were identified by MS/MS analysis and the MASCOT score is indicated.

### Comparative Proteomic Analysis between the Vesicular and Micellar Phases

**Figure 3:**
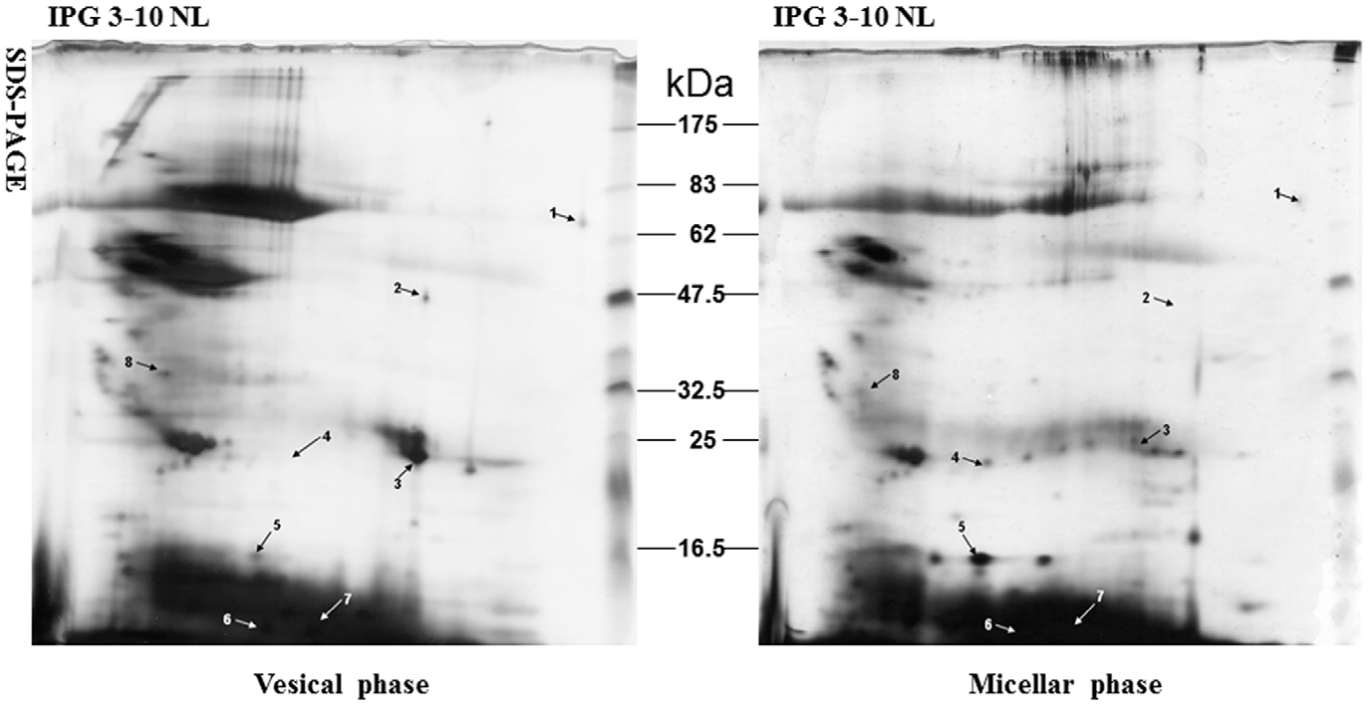
Representative 2-DE pattern of gallbladder bile proteins from different phases of cholesterol-phosphatide vesicle and micelle. The proteins (250 µg) were subjected to 2-DE system (first dimension, IPG strip, pH 3–10 NL, 13 cm; second dimension, 12% SDS-PAGE). Protein spots were visualized by silver staining and analyzed by ImageMaster™ 2-D Platinum software. The spots marked with arrows in the two images indicate the distribution of up-regulated or down-regulated proteins that were successfully identified by MALDI-TOF. The numbers between the two images show the molecular marker (kDa). Annotations in the gels refer to the Spot No. are in Table 3.

There were approximately 120±24 (n = 5) spots detected on vesicular phase gels and 198±37 (n = 5) on micellar phase gels. There were 72±16 (45.3%) spots matching vesicular with micellar phases. Compared with the micellar phase, 8 spots we focused, which were identified successfully by MS, displayed differentially expressed in vesicular phase (Figure 3).

**Table 3:** Differentially expressed proteins between vesicular phase and control micellar phase identified by MALDI-TOF MS.

SpotNo.a)	Protein Descriptionb)	Accession No.c)	Theoretical Mr (Da)/pId)	Matched peptidese)	Protein coverage (%)f)	Protein Scoreg)
1	Chain A, Crystal structure of Human Serum Albumin	3212456	68424.7/5.67	39	64	656
2	Aldehyde Reductase	1633300	36418.8/6.34	18	49	336
3	Beta Globin	71727231	15987.3/7.86	9	61	208
4	Apolipoprotein A-I, Isoform CRA_b	119587681	23454/5.39	15	57	223
5	Apolipoprotein A-II, Precursor	4502149	11175/5.05	6	69	233
6	Chain A, Prealbumin (Human Plasma)	230651	13808.9/5.55	5	32	79
7	Chain A, Human Retinol Binding Protein With Its Carrier Protein Transthyretin Reve	4558175	13454.7/5.5	8	69	344
8	Haptoglobin Beta Chain	998962	26275.1/6.32	10	43	157

a) Spot number corresponds to the spot number on Fig. 3.

b) Protein description refers to the name of each matched protein in NCBInr database.

c) Accession number is recorded as a reference for the identification in NCBInr database.

d) Theoretical Mr/pI means theoretical molecular weight and iso-electric point of the matched protein.

e) Matched peptide refers to the number of peptide matched to the candidate protein.

f) Sequence coverage is percent of identified sequence to the complete sequence of the candidate protein.

g) Spots were identified by MS/MS analysis and the MASCOT score is indicated.

### PMF and MALDI-TOF MS Identification of the Differentially Expressed Proteins

The extracted peptides from these focused spots were identified by MALDI-TOF MS. The protein description, accession number, theoretical molecular weight and iso-electric point, peptides matched, coverage and scores of altered expressed protein spots were presented in Table 2 referred to Figure 2 and Table 3 referred to Figure 3. Several proteins were present as multiple spots on 2-D gels in Figure 2, which might represent different post-translational modification that alter the molecular mass or pI of the protein. For example, spot 12 and 19 were identified as lipid-free human Apolipoprotein A-I (NCBInr, theoretical MW/pI 28061.5 Da/5.27). All identified proteins listed in Table 2 and 2 were classified into functional groups including transporter, defenses immunity, structural molecule, enzyme, unknown proteins, etc., based on their functional categories in the “SWISS-PROT and TrEMBL” protein database.

### Validation of Differentially Expressed Proteins by Western Blot Analysis

The expression level of HSA and RBP were up-regulated in cholesterol cholelithiasis group and vesicular phase. However, the PFN was significant lower in cholesterol cholelithiasis group than in control group. The expression profiles of the three proteins were consistent with 2-DE results. These results validated the reliability of proteomic analysis as described above.

## Discussion

The mechanism of the formation of cholesterol gallstone remains unclear. The role of nucleating proteins, in the process of cholesterol crystallization, became increasingly controversial. Some publications confirmed that most biliary proteins are currently considered to be secondary to gallstone formation or inflammation, with the possible exception of mucin [11], [12]. However, other scientists reach a consensus that the biliary proteins might play an important role [23]. In general, in vivo studies that analyzed the role of putative pro-nucleating proteins have been hampered by a number of factors. For example, prior methods used to identify quantitative differences in these biliary proteins were relatively insensitive and might be inadequate to demonstrate significant differences in concentration. Not the concentrations of, but rather accumulative or cooperative effect of multiple proteins could be required to promote the formation of cholesterol gallstones. Utilization of sensitive proteomic screens techniques will likely increase our understanding of what role, if any, the biliary proteins may play in the course of formation of cholesterol gallstones [24].

Various cholesterol-carried phases were isolated using the density gradient centrifugation method in the study, which was first described by Amigo et al in 1990 [25]. And this method was improved by Miquel et al in 1992 [19]. Subsequently, some researchers argued that there are artificial shifts between vesicular and micellar phases during the procedure [26]. Accordingly, gel filtration of bile using an eluent containing bile salts should theoretically allow isolation of vesicles and micelles without artificial perturbation. However, the gel filtration may underestimate vesicular lipids and overestimate vesicular cholesterol phospholipid ratios [27]. Ultracentrifugation perhaps has some advantages, such as vesicles being isolated in a reproducible method with relatively short time and harvested from the original bile specimen without the addition of exogenous bile salts or the diluted effect represented by the volume of elution buffers used for column chromatography. In our opinion, the optimal methodology for quantification of cholesterol carriers is combined ultracentrifugation-ultrafiltration-dialysis described by Moschetta et al [28].

Compared with previous results, the separated proteins in our 2-DE maps are sufficient and most of them are also identified in their experiments (HSA, Haptoglobin, Prealbumin, and so on). Moreover, several proteins are only identified in our experiment (PFN, Aldo-keto Reductase Family 1 member A1, etc.).

The differentially expressed proteins had not been identified in the past studies. In this experiment, we compared the proteomes between two groups or two phases by 2-DE and MALDI-TOF MS. Finally, 22 and 8 protein spots were identified, respectively. Five of them were identical (Apolipoprotein A-I, Apolipoprotein A-II, HSA, Prealbumin and RBP). At the same time, three interested proteins (HSA, PFN and RBP) were validated by Western blot. The results were consistent between 2-DE and Western blot, which confirmed the validity of the comparative proteomic study.

Based on their biological function, the differentially expressed proteins can be classified into transport proteins (HSA, Beta-globin, Hemoglobin, Transthyretin), enzymes or enzyme regulator (Aldo-keto Reductase, Aldehyde Reductase), immunity system (Immunoglobulin Heavy Chain, Alpha1-acid Glycoprotein), structure molecule (Beta-actin, Tropomyosin), proteins in the coagulation cascade (Fibrinogen Beta Chain Precursor), and so on. Some identified proteins are of particular interest and discussed briefly here.

HSA, the main protein of plasma, has a good binding capacity for calcium ion, fatty acids, hormones, bilirubin and drugs. The expressional level is more than three-fold higher in cholesterol gallstone than in control group, and more than two-fold higher in vesicular than in micellar phase. The relationship of HSA with the cholesterol gallstone remains unclear. The up-regulated albumin could possibly be a consequence of cholesterol cholelithiasis. Keulemans et al [29] discovered that albumin, IgG, aminopeptidase N, and mucin were selectively increased in 36% of bile samples. Correspondingly, they hypothesized that local inflammatory processes were the primary factor, which led to an increase in mucin and IgG production. Then these factors might trap albumin via hydrophobic interaction. However, HSA identified in present experiment is modified, which could be a cause of the cholesterol gallstone [30]. More recent studies have uncovered a myriad of pathobiologic events induced by modified albumin [31], [32]. Therefore, it may perform function in the course of disturbance of bile thermodynamic or dynamics equilibrium through certain mechanism, which should be further studied.

The expressional abundance of RBP is more than three-fold in cholesterol gallstone than control groups. The same results appeared in different cholesterol-carried phases. RBP is synthesized primarily in the liver, delivering retinol from the liver stores to various target tissues. There are no literatures reported about the relationship of RBP and cholesterol cholelithiasis. The result indicates that RBP probably plays a certain role in the formation of cholesterol gallstone, which is now investigated in native and model bile in our group.

PFN is a ubiquitous protein found in many types of eukaryotic systems and plays a role both in signal transduction pathways and in actin filament dynamics [33]. In mammalian tumor cells, over-expression of PFN led to suppression of tumorigenicity and cell malignant behavior, such as abnormal cell proliferation, spreading, adhesion, and poor differentiation [34]. Therefore, we predict that down-regulated PFN might lead to the gallbladder motor dysfunction, which facilitates the cholesterol monohydrate crystal formation in the cholesterol supersaturated bile.

Among the up-regulated proteins, Calreticulin is a calcium-binding chaperone in the ER, which participates in the folding of newly synthesized proteins and glycoproteins [35]. Over-expression Calreticulin indicated that glycoproteins synthesis is increasing in cholesterol gallstone patients. Most of pro-nucleating agents belonging to glycoproteins, Calreticulin may enhance the formation of cholesterol gallstone by promoting the expression of glycoproteins.

The differentially expressed proteins were compared with the previous studies of cholesterol gallstone formation. Some were found to involve in the mechanism of formation of gallstones before. Among the up-regulated proteins in gallstone group or vesicular phase, Immunoglobulin, Alpha-1 acid Glycoprotein and Haptoglobin are considered to be pro-nucleating agents. Immunoglobulin might promote nucleation by binding to vesicle phospholipids and causing vesicles to aggregate, or by acting as auto-antibodies against anti-nucleating substances [36]. Abei et al [37] reported that removal of alpha-1 acid glycoprotein reduced the pro-nucleating effect of the Con-A binding fraction from human bile by 33%. Yamashita et al [38] reported that biliary Haptoglobin at its physiological concentration had a highly potent crystallization promoting activity.

Among the down-regulated proteins, Apolipoprotein A-I and Apolipoprotein A-II were considered to be putative anti-nucleation factors in the previous reports. There were some hydrophobic regions in these proteins that might interact with lipid molecules. Kibe et al [10] discovered that these proteins prolonged the nucleation time of cholesterol monohydrate crystals when added to model systems of supersaturated bile at physiological concentrations. Furthermore, they were among the proteins present in a fraction of bile enriched in potent inhibitors of cholesterol crystal nucleation.

Most of these studies, focused on the role of pro-nucleating and anti-nucleating proteins in the process of cholesterol crystallization, were performed in the eighties en nineties of the previous century. Their nucleating roles became increasingly controversial. Mucin, Immunoglobulins and some other glycoproteins were regarded as pro-nucleating proteins. Apolipoprotein A-I and Apolipoprotein A-II were considered to be putative anti-nucleation factors. These conclusions were based on different methods, including polarizing microscopy, nephelometry, or spectrophotometry. However, crystal mass will be underestimated for large crystals due to morphology and size of cholesterol crystals. Chemical measurement of crystal mass should be regarded as the ideal method for quantitation of cholesterol crystallization when it was investigated that the role of nucleation proteins during the course of cholesterol gallstone formation [39]. Large-scale, sensitive proteomic screens are becoming widely used in pathophysiology and translational research. Comparative proteomic techniques will likely improve our understanding of what role, if any, biliary proteins may play in formation of cholesterol gallstones [24]. It remains controversial that the differentially expressed proteins, discovered in our study, are primary roles in cholesterol gallstone formation, or secondary to gallstone formation or inflammation. Therefore, the accurate roles of the differential proteins in the formation of cholesterol gallstone should be further investigated.

Some differentially expressed proteins, Beta-actin, PFN, Tropomyosin, may affect the gallbladder motility, which is also crucial prerequisite for cholesterol gallstone formation. It remains indistinct that other differentially expressed proteins, such as RBP, HSA, Calreticulin, Fibrinogen and Aldo-keto Reductase, are causes or consequences of the cholesterol gallstone. It may be possible to select some proteins according to their character and biological function for further investigation of their roles in the formation of cholesterol gallstones.

In conclusion, based on 2-DE, MALDI-TOF MS and tandem TOF/TOF MS, we made an attempt to clarify the differentially expressed proteins between the cholesterol gallstone and control groups, and between the vesicular and micellar phases. The present results about some of the differentially expressed proteins involved in the gallstone formation are in good agreement with previous data. Other proteins may be involved in the formation of cholesterol gallstone through particular mechanisms. The information from this study may be helpful to provide some important clues to uncover the pathophysiologic molecular mechanisms involved in the formation of cholesterol cholelithiasis. However, further studies are still needed to elucidate the detailed roles of these differentially expressed proteins.

## References

[1] Marschall HU, Einarsson C (2007) Gallstone disease. Journal of Internal Medicine 261: 529–542. 17547709 10.1111/j.1365-2796.2007.01783.x

[2] Sandler RS, Everhart JE, Donowitz M, Adams E, Cronin K, et al (2002) The burden of selected digestive diseases in the United States. Gastroenterology 122: 1500–1511. 11984534 10.1053/gast.2002.32978

[3] Portincasa P, Moschetta A, Palasciano G (2006) Cholesterol gallstone disease. Lancet 368: 230–239. 16844493 10.1016/S0140-6736(06)69044-2

[4] Diehl AK (1991) Epidemiology and Natural-History of Gallstone Disease. Gastroenterology Clinics of North America 20: 1–19. 2022415

[5] Wang H, Portincasa P, Liu M, Tso P, Samuelson L, et al (2010) Effect of gallbladder hypomotility on cholesterol crystallization and growth in CCK-deficient mice. Biochim Biophys Acta 180: 138–146. 10.1016/j.bbalip.2009.10.003 PMC2830894 19836465

[6] Venneman NG, Portincasa P, van Berge-Henegouwen GP, van Erpecum KJ (2004) Cholesterol saturation rather than phospholipid/bile salt ratio or protein content affects crystallization sequences in human gallbladder bile. European Journal of Clinical Investigation 34: 656–663. 15473890 10.1111/j.1365-2362.2004.01409.x

[7] Sömjen G, Gilat T (1985) Contribution of vesicular and micellar carriers to cholesterol transport in human bile. Journal of Lipid Research 26: 699–704. 4031648

[8] Burnstein M, Ilson R, Petrunka C, Taylor R, Strasberg S (1983) Evidence for a potent nucleating factor in the gallbladder bile of patients with cholesterol gallstones. Gastroenterology 85: 801–807. 6884705

[9] Harvey PRC, Upadhya GA, Strasberg SM (1991) Immunoglobulins as Nucleating Proteins in the Gallbladder Bile of Patients with Cholesterol Gallstones. Journal of Biological Chemistry 266: 13996–14003. 1856228

[10] Kibe A, Holzbach R, LaRusso N, Mao S (1984) Inhibition of cholesterol crystal formation by apolipoproteins in supersaturated model bile. Science 225: 514–516. 6429856 10.1126/science.6429856

[11] Miquel JF, Núñez L, Amigo L, González S, Raddatz A, et al (1998) Cholesterol saturation, not proteins or cholecystitis, is critical for crystal formation in human gallbladder bile. Gastroenterology 114: 1016–1023. 9558292 10.1016/s0016-5085(98)70322-1

[12] Wang DQ, Cohen DE, Lammert F, Carey MC (1999) No pathophysiologic relationship of soluble biliary proteins to cholesterol crystallization in human bile. Journal of Lipid Research 40: 415–425. 10064729

[13] Jirsa M, Groen AK (2001) Role of biliary proteins and non-protein factors in kinetics of cholesterol crystallisation and gallstone growth. Frontiers in Bioscience 6: E154–E167. 11689352 10.2741/jirsa

[14] He C, Jüngst D (1996) Electrophoretic analysis of biliary proteins: Application of high resolution two-dimensional polyacrylamide gel electrophoresis with immobilized pH gradient in the first dimension. Electrophoresis 17: 617–619. 8740188 10.1002/elps.1150170334

[15] He C, Fischer S, Meyer G, Muller I, Jungst D (1997) Two-dimensional electrophoretic analysis of vesicular and micellar proteins of gallbladder bile. Journal of Chromatography A 776: 109–115. 9286084 10.1016/s0021-9673(97)00560-8

[16] Kristiansen TZ, Bunkenborg J, Gronborg M, Molina H, Thuluvath PJ, et al (2004) A proteomic analysis of human bile. Molecular & Cellular Proteomics 3: 715–728. 15084671 10.1074/mcp.M400015-MCP200

[17] Zhou H, Chen B, Li RX, Sheng QH, Li SJ, et al (2005) Large-scale identification of human biliary proteins from a cholesterol stone patient using a proteomic approach. Rapid Communications in Mass Spectrometry 19: 3569–3578. 16276486 10.1002/rcm.2207

[18] Strasberg SM, Harvey PRC, Hofmann AF (1990) Bile Sampling, Processing and Analysis in Clinical-Studies. Hepatology 12: S176–S182. 2210646

[19] Miquel JF, Rigotti A, Rojas E, Brandan E, Nervi F (1992) Isolation and Purification of Human Biliary Vesicles with Potent Cholesterol-Nucleation-Promoting Activity. Clinical Science 82: 175–180. 1311655 10.1042/cs0820175

[20] Mortz E, Krogh TN, Vorum H, Görg A (2001) Improved silver staining protocols for high sensitivity protein identification using matrix-assisted laser desorption/ionization-time of flight analysis. Proteomics 1: 1359–1363. 11922595 10.1002/1615-9861(200111)1:11<1359::AID-PROT1359>3.0.CO;2-Q

[21] Xu ZB, Zhou XW, Lu HJ, Wu N, Zhao HB, et al (2007) Comparative glycoproteomics based on lectins affinity capture of N-linked glycoproteins from human Chang liver cells and MHCC97-H cells. Proteomics 7: 2358–2370. 17623300 10.1002/pmic.200600041

[22] Görg A, Obermaier C, Boguth G, Harder A, Scheibe B, et al (2000) The current state of two-dimensional electrophoresis with immobilized pH gradients. Electrophoresis 21: 1037–1043. 10786879 10.1002/(SICI)1522-2683(20000401)21:6<1037::AID-ELPS1037>3.0.CO;2-V

[23] Miquel JF, van der Putten J, Pimentel F, Mok KS, Groen AK (2001) Increased activity in the biliary Con A-binding fraction accounts for the difference in crystallization behavior in bile from Chilean gallstone patients compared with Dutch gallstone patients. Hepatology 33: 328–332. 11172333 10.1053/jhep.2001.21550

[24] Maurer KJ, Carey MC, Fox JG (2009) Roles of infection, inflammation, and the immune system in cholesterol gallstone formation. Gastroenterology 136: 425–440. 19109959 10.1053/j.gastro.2008.12.031 PMC2774219

[25] Amigo L, Covarrubias C, Nervi F (1990) Rapid Isolation of Vesicular and Micellar Carriers of Biliary Lipids by Ultracentrifugation. Journal of Lipid Research 31: 341–347. 2324652

[26] Yuet PK, Blankschtein D, Donovan JM (1996) Ultracentrifugation systematically overestimates vesicular cholesterol levels in bile. Hepatology 23: 896–903. 8666347 10.1002/hep.510230434

[27] Donovan JM, Jackson AA (1998) Accurate separation of biliary lipid aggregates requires the correct intermixed micellar/intervesicular bile salt concentration. Hepatology 27: 641–648. 9500688 10.1002/hep.510270301

[28] Moschetta A, Eckhardt ER, De Smet MB, Renooij W, Van Berge-Henegouwen GP, et al (2001) Accurate separation of vesicles, micelles and cholesterol crystals in supersaturated model biles by ultracentrifugation, ultrafiltration and dialysis. Biochim Biophys Acta 1532: 15–27. 11420170 10.1016/s1388-1981(01)00110-x

[29] Keulemans YCA, Mok KS, de Wit LT, Gouma DJ, Groen AK (1998) Hepatic bile versus gallbladder bile: A comparison of protein and lipid concentration and composition in cholesterol gallstone patients. Hepatology 28: 11–16. 9657090 10.1002/hep.510280103

[30] Grattagliano I, Wang D, Di Ciaula A, Diogo C, Palasciano G, et al (2009) Biliary proteins and their redox status changes in gallstone patients. European Journal of Clinical Investigation 39: 986–992. 19656169 10.1111/j.1365-2362.2009.02187.x

[31] Hattori Y, Suzuki M, Hattori S, Kasai K (2002) Vascular Smooth Muscle Cell Activation by Glycated Albumin (Amadori Adducts). Hypertension 39: 22–29. 11799073 10.1161/hy1201.097300

[32] Cohen MP (2003) Intervention strategies to prevent pathogenetic effects of glycated albumin. Archives of Biochemistry and Biophysics 419: 25–30. 14568005 10.1016/j.abb.2003.08.012

[33] Yarmola EG, Bubb MR (2006) Profilin: emerging concepts and lingering misconceptions. Trends in Biochemical Sciences 31: 197–205. 16542844 10.1016/j.tibs.2006.02.006

[34] Janke J, Schluter K, Jandrig B, Theile M, Kolble K, et al (2000) Suppression of tumorigenicity in breast cancer cells by the microfilament protein profilin 1. Journal of Experimental Medicine 191: 1675–1685. 10811861 10.1084/jem.191.10.1675 PMC2193149

[35] Williams DB (2006) Beyond lectins: the calnexin/calreticulin chaperone system of the endoplasmic reticulum. Journal of Cell Science 119: 615–623. 16467570 10.1242/jcs.02856

[36] Oleary DP (1995) Biliary Cholesterol Transport and the Nucleation Defect in Cholesterol Gallstone Formation. Journal of Hepatology 22: 239–246. 7790713 10.1016/0168-8278(95)80435-8

[37] Abei M, Schwarzendrube J, Nuutinen H, Broughan TA, Kawczak P, et al (1993) Cholesterol Crystallization-Promoters in Human Bile - Comparative Potencies of Immunoglobulins, Alpha(1)-Acid Glycoprotein, Phospholipase-C, and Aminopeptidase-N. Journal of Lipid Research 34: 1141–1148. 8103787

[38] Yamashita G, Corradini SG, Secknus R, Takabayashi A, Williams C, et al (1995) Biliary Haptoglobin, a Potent Promoter of Cholesterol Crystallization at Physiological Concentrations. Journal of Lipid Research 36: 1325–1333. 7666009

[39] Portincasa P, Venneman NG, Moschetta A, van den Berg A, Palasciano G, et al (2002) Quantitation of cholesterol crystallization from supersaturated model bile. Journal of Lipid Research 43: 604–610. 11907143

